# Editorial: Insights into lipid biology and function in insect systems

**DOI:** 10.3389/finsc.2022.1119577

**Published:** 2022-12-22

**Authors:** Geoffrey Michael Attardo, Immo Alex Hansen

**Affiliations:** ^1^ Department of Entomology and Nemotology, College of Agricultural and Environmental Sciences, University of California, Davis, Davis, CA, United States; ^2^ Department of Biology, New Mexico State University, Las Cruces, NM, United States

**Keywords:** lipid, insect, metabolism, fat body, energy

Lipids, in their various permutations, act as one of the fundamental building blocks of all life on earth. Lipids are probably best known for their role as high-density energy storage and transport molecules; however, they also fulfill an array of essential organismal functions. They act as the key constituents in cellular membranes providing an adaptable interface between cells and their surroundings. The lipid bilayer facilitates structural and functional capabilities required for cellular specialization and rapid responses to environmental changes (1). They also function as signaling molecules critical for maintaining nutritional homeostasis (2) and acting as immunomodulators regulating inflammatory and cellular responses to invading organisms (3).

The tremendous diversity in terms of structure and function associated with insects reveals fascinating specializations in the use of lipids to facilitate their life histories (4, 5). Lipids are precursors for the production of many insect hormones (6, 7). Waxes strengthen and waterproof insect cuticles and eggs to protect against damage/desiccation and coat insect tracheole systems to facilitate breathing in terrestrial and aquatic insects (8, 9). The mechanisms associated with the synthesis, storage, mobilization, and metabolism of lipids in insects are similar to that of vertebrate animals (10). However, the context in which these activities are performed differs depending on the insect’s life history. The study of lipid biology in insects provides new insights into fundamental mechanisms of lipid biochemistry and identifies novel mechanisms and uses of lipids within the context of highly specialized physiological functions. This collection features four articles covering different aspects of lipid biology in insects.

In Pinch et al. “*Fat and Happy: Profiling Mosquito Fat Body Lipid Storage and Composition Post-blood Meal*” the authors characterize the dynamics of the lipidome over the course of vitellogenesis in the Yellow Fever mosquito *Aedes aegypti*. Vitellogenesis represents the digestion of nutrients derived from a vertebrate blood meal and the activation of oogenesis. This process is regulated by a complex series of nutritional and hormonal signals and functions to transfer nutrients to developing oocytes. The processes of blood meal digestion, nutrient transfer, and oocyte development are lipid-intensive processes. This study provides a comprehensive analysis of the tissue morphology (digestive tract and ovaries) and lipidomic changes over the course of vitellogenesis. The study offers an array of insights into lipid-associated metabolic processes during vitellogenesis. It opens the way for new investigations into the regulation of nutrient transfer during this intensive and dynamic process.

In Aredes et al., “*Silencing of Mitochondrial Trifunctional Protein A Subunit (HADHA) Increases Lipid Stores, and Reduces Oviposition and Flight Capacity in the Vector Insect Rhodnius prolixus*” the authors investigate the function of the protein Mitochondrial Trifunctional Protein A subunit in regulating the utilization of nutritional lipids by the Chagas disease vector *Rhodnius prolixus.* The MTP protein complex plays a critical role in the β-oxidation of fatty acids within the mitochondria. The authors show that the gene for this protein responds in a tissue-specific manner to blood feeding and regulates lipid metabolism required for vital physiological processes, including flight and oogenesis.

In the review, “*Roles of Insect Oenocytes in Physiology and Their Relevance to Human Metabolic Diseases*” Huang et al. provide a comprehensive overview of the function of oenocyte cells in the fat body tissue and how their function in insects relates to metabolic disease in humans. The paper highlights the parallels between oenocyte function and liver hepatocytes in mammals. They highlight the role of these cells in regulating lipid storage, synthesis, metabolism, and mobilization. Oenocytes appear to undergo significant crosstalk with other cells/organs to regulate lipid homeostasis and are responsive to nutrient-based signaling. These cells also play roles in detoxification and immune responses. This review provides important insights into these understudied cells. It reveals important parallels between insects and mammals regarding the conservation of function in lipid metabolism, homeostasis, immunity, and detoxification. These insights highlight these cells’ essential role in insect physiology and emphasize the power that insect-based experimental model systems can bring to our understanding of metabolic diseases in vertebrate systems.

Finally, the review by Gade and Marco, “*The Adipokinetic Peptides of Hemiptera: Structure, Function, and Evolutionary Trends*”, provides a comprehensive overview of the structure, function, and evolution of adipokinetic hormone in the Hemiptera, a species-rich and highly diverse insect order. The paper explores the members of this order, many of which function as important vectors of plant and animal diseases, and describes the amazing diversity of function and life histories found within. This diversity provides a complex array of life history specializations requiring specialized regulation of lipid and carbohydrate metabolism mediated by the peptide hormone AKH. The authors provide a historical summary of the research determining the structure and function of AKH hormones in Hemiptera and examine the changes in AKH structure over the course of hemipteran evolution. They propose a model of AKH peptide evolution in the Hemiptera and highlight the potential for using species-specific hormone analogs to develop target-specific “green insecticides.”

This Research Topic brings together a fascinating collection of findings and insights into the dynamics, mechanics, physiology, and evolution of lipid biology in insects. The development of new analytical technologies facilitates new avenues of research on the biology of lipids. It allows investigators to explore these areas in greater depth and detail and raises the prospect of exciting discoveries in this field at basic and applied levels. We want to thank the authors for their valued contributions to this important and growing field of insect research.

## Author contributions

All authors listed have made a substantial, direct, and intellectual contribution to the work and approved it for publication.
